# An Evaluation of Putative Sympatric Speciation within *Limnanthes* (Limnanthaceae)

**DOI:** 10.1371/journal.pone.0036480

**Published:** 2012-05-01

**Authors:** Stephen C. Meyers, Aaron Liston, Robert Meinke

**Affiliations:** Department of Botany and Plant Pathology, Oregon State University, Corvallis, Oregon, United States of America; Instituto de Higiene e Medicina Tropical, Portugal

## Abstract

*Limnanthes floccosa* ssp. *floccosa* and *L. floccosa* ssp. *grandiflora* are two of five subspecies within *Limnanthes floccosa* endemic to vernal pools in southern Oregon and northern California. Three seasons of monitoring natural populations have quantified that *L. floccosa* ssp. *grandiflora* is always found growing sympatrically with *L. floccosa* ssp. *floccosa* and that their flowering times overlap considerably. Despite their subspecific rank within the same species crossing experiments have confirmed that their F1 hybrids are sterile. An analysis of twelve microsatellite markers, with unique alleles in each taxon, also shows exceedingly low levels of gene flow between populations of the two subspecies. Due to the lack of previous phylogenetic resolution among *L. floccosa* subspecies, we used Illumina next generation sequencing to identify single nucleotide polymorphisms from genomic DNA libraries of *L. floccosa* ssp. *floccosa* and *L. floccosa* ssp. *grandiflora*. These data were used to identify single nucleotide polymorphisms in the chloroplast, mitochondrial, and nuclear genomes. From these variable loci, a total of 2772 bp was obtained using Sanger sequencing of ten individuals representing all subspecies of *L. floccosa* and an outgroup. The resulting phylogenetic reconstruction was fully resolved. Our results indicate that although *L. floccosa* ssp. *floccosa* and *L. floccosa* ssp. *grandiflora* are closely related, they are not sister taxa and therefore likely did not diverge as a result of a sympatric speciation event.

## Introduction

With the notable exception of polyploid speciation in plants, sympatric speciation, the theory that genetic divergence within an interbreeding population can result in the evolution of new species, remains an intensely debated issue [1], [2]. In contrast, allopatric speciation, in which new species arise as a result of geographic isolation, is uncontroversial with numerous observed and experimental examples.

First proposed by Darwin [3], sympatric speciation held a theoretically prominent, or at least equal role, compared to allopatric speciation until the “neo-Dawinian synthesis” of the mid twentieth century. During that period of time, prominent evolutionary biologists such as Dobzhansky [4] and Mayr [5], [6] strongly dismissed sympatric speciation as unlikely or very uncommon. Due in large part to their work, allopatric speciation has become considered the null hypothesis of most speciation events. However, with foresight Mayr predicted “The issue will be raised again at regular intervals. Sympatric speciation is like the Lernaean Hydra which grew two new heads whenever one of its old heads was cut off “ [6].

Indeed, in part, as a result of the increased use of molecular phylogenetic techniques within recent decades, many evolutionary biologists have challenged this orthodoxy based on empirical evidence [7] and one experimental study [8]. Additionally, numerous theoretical models have posited that divergent selection may be able to surmount recombination in order to disrupt Hardy-Weinberg equilibrium, therefore sympatric speciation may be more common than traditionally assumed [1], [7].

Due to the growing number of putative examples of sympatric speciation, as well as the widely varying quality and quantity of criteria used to ascertain a speciation event, Coyne and Orr [1] have proposed four rigorous criteria that they conclude must be met in order to reject an allopatric speciation null hypothesis. These are, which we modify slightly:

1. *The species must be largely or completely sympatric.* Here, Coyne and Orr are stipulating that individuals of the species in question must physically occur within the dispersal distance (or cruising range) of one another.
2. *The species must have substantial reproductive isolation.* In other words, speciation must be complete. Evaluating this criterion can be subjective because of the numerous and often conflicting species concepts. As a result, we chose to evaluate this criterion using the least subjective of the species concepts, namely the biological species concept.
3. *The sympatric taxa must be sister groups.* Preferably, this criterion should be evaluated using multiple loci. This is due to the fact that hybridization may falsely indicate, at a single locus, a sister relationship between two species, which in reality, are not closely related. In practice, an evaluation of molecular data from organelles and multiple nuclear loci may often be necessary in order to insure an accurate phylogeny.
4. *The biogeographic and evolutionary history of the groups must make the existence of an allopatric phase very unlikely*.”

Since their publication, two prominent studies have been presented in which the authors claim to have satisfied all of Coyne and Orr's criteria [9], [10]. These studies, however, have been criticized for lack of rigor in meeting at least one of the four criteria. For example, the Barluenga et al. study [9] involving cichlid species in a Nicaraguan crater lake, has been criticized for incomplete taxon sampling [11].

The second study [10], which hypothesizes a sympatric speciation event associated with two palm species on Lord Howe Island, has been questioned as a result of the authors' failure to take into account the geological history of the island. Stuessy [12] argues, that in consideration of the fact that Lord Howe Island is currently approximately 5% of its original size, an allopatric speciation event is more parsimonious. Additionally, in a later paper, some of the original authors of the Lord Howe Island study have suggested that this speciation event may be more accurately referred to as parapatric [13].

In this study, using Coyne and Orr's criteria as a guideline, we surveyed the potential of a sympatric speciation event involving two subspecies of *Limnanthes floccosa*, which co-occur near vernal pools in southwest Oregon, USA. More strictly, we chose to study the evolutionary relationship between *Limnanthes floccosa* ssp. *floccosa* and *L. floccosa* ssp. *grandiflora*. Because these taxa show complete reproductive isolation (see below) we refer to them as species in the subsequent text.

**Figure 1:**
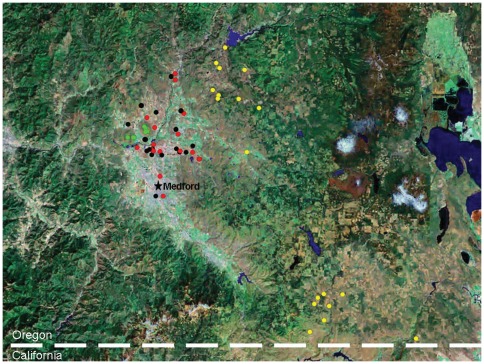
Locations of current and historic populations of *L. f.* ssp. *floccosa*. Black; *L. f.* ssp. *bellingeriana*, yellow; *L. f.* ssp. *grandiflora*, red and *L. f.* ssp. *pumila*, green. (Courtesy of the Oregon Flora Project).

Although no previous authors have specifically suggested this species pair as an example of sympatric speciation, these taxa were chosen as having high potential to meet Coyne and Orr's criteria, based on the following reasons:

1. All known populations of *L. floccosa* ssp. *grandiflora* have been described as occurring within close physical proximity to populations of *L. floccosa* ssp. floccosa [14]. Furthermore, within *L. floccosa*, only *L. floccosa* ssp. *floccosa* and *L. floccosa* ssp. *grandiflora* are known to co-occur within the same vernal pools (Figure 1).
2. The breeding system of *L. floccosa* ssp. *floccosa* is predominantly selfing, while *L. floccosa* ssp. *grandiflora* is partially autogamous (approximately 50% outcrossing and 50% selfing) [15]. We hypothesized that this mixture of breeding systems may indicate a high or complete level of reproductive isolation between the two taxa.
3. *Limnanthes floccosa* ssp. *floccosa* and *L. floccosa* ssp. *grandiflora* have, in a previous study, been shown to be closely related and were suggested to be sister taxa [16]. However, in this study phylogenetic relationships within *L. floccosa* were unresolved. Furthermore, both subspecies are diploid and have the same chromosome number (*n* = 5).
4. *Limnanthes floccosa* ssp. *floccosa* and *L. floccosa* ssp. *grandiflora* are vernal pool taxa. This environment has been suggested as having high potential for speciation within genera adapted to this particular habitat [17].
5. *Limnanthes* species are annuals and easily grown in a greenhouse environment. As such, unlike many animal and perennial plant species, they are amenable to experimentation, such as a crossing study.

Our strategy, in order to further address the potential of a sympatric speciation event involving this species pair, was to conduct a multi-year spatial and temporal monitoring assessment of *L. floccosa* ssp. *floccosa* and *L.* ssp. *floccosa* ssp. *grandiflora* populations. Additionally, through a microsatellite study and a greenhouse hybridization experiment, we investigated the amount of reproductive isolation between the two taxa. Lastly, we used next generation (Illumina) technology to sequence genomic DNA libraries of *L. floccosa* ssp. *floccosa* and *L. floccosa* ssp. *grandiflora* to identify SNPs (single nucleotide polymorphisms) within the nuclear, chloroplast and mitochondria genomes. In turn, these data were used to reconstruct a fully resolved phylogeny of *L. floccosa*.

## Results

### Spatial and temporal sympatry

Although, on average, *L. floccosa* ssp. *floccosa* plants were found at a slightly further distance from the vernal pools than *L. floccosa* ssp. *grandiflora* plants (1.45 m versus 1.22 m), in general the two species have a largely overlapping habitat preference (Figure S1). In most instances, *L. floccosa* ssp. *grandiflora* plants are found less than one meter from *L. floccosa* ssp. *floccosa* plants. Equivalently, while the bud, flower and seed timing of some *L. floccosa* ssp. *grandiflora* plants occurs slightly earlier than those of *L. floccosa* ssp. *floccosa*, the two taxa are predominantly temporally sympatric (Figure S2).

### Reproductive isolation

As a result of the 80 *L. floccosa* ssp. *floccosa*×*L. floccosa* ssp. *grandiflora* crosses 269 seeds were produced. From among those seeds 30 (11%) successfully germinated. Of the successful germinates, 21 plants were depauperate in morphology and died within one to three weeks of germination. All nine plants that survived to maturity were sterile and produced no seeds (Table S1).

Of the 240 plants of *L. floccosa* ssp. *floccosa* and *L. floccosa* ssp. *grandiflora*, collected in the wild from three sites, the microsatellite survey indicated that only one *L. floccosa* ssp. *grandiflora* specimen shared an allele, at one locus, with *L. floccosa* ssp. *floccosa* (Table S2). All remaining specimens showed fixed (or null) alleles.

Among the eight surviving hybrid *L. floccosa* ssp. *floccosa*×*L. floccosa* ssp. *grandiflora* plants, produced in the greenhouse study, all were heterozygous at some loci, displaying both *L. floccosa* ssp. *floccosa* and *L. floccosa* ssp. *grandiflora* alleles at eight of the twelve loci. At the four remaining loci, all plants were homozygous for the allele of the maternal parent of the cross, indicating uniparental inheritance.

### Phylogeny

After removal of the 4 bp 5′ tag, Illumina sequencing resulted in approximately 5,000,000 72 bp paired end microreads per species (Table S3). The *de novo* assembly of the microreads resulted in nearly 400 contigs, ranging in size from less than 100 bp to approximately 48,000 bp for each species. Further alignment of the ∼400 contigs to the *C. papaya* complete chloroplast genome resulted in the assembly of ∼95% of the *L. floccosa* ssp. *grandiflora* chloroplast genome. This alignment was composed of six contigs ranging in size from approximately 48 kbp to 400 bp. Secondary alignments, using the *Carica- L. floccosa* ssp. *grandiflora* chimeric pseudo-reference generated nearly complete (∼99%) *L. floccosa* ssp. *floccosa* and *L. floccosa* ssp. *grandiflora* chloroplast genomes. Sanger sequencing of all remaining gaps, areas with possible long lengths of repetitive DNA and putative SNPs resulted in the assembly of similar length chloroplast genomes for *L. floccosa* ssp. *floccosa* and *L. floccosa* ssp. *grandiflora* (152, 357 and 152,355 bp respectively; Genbank accession numbers HQ179768, HQ179769). Average coverage of these genomes was over 300× (Table S4).

Approximately 85% of the *L. alba* chloroplast genome (132,564 bp) was assembled from the *L. alba* genome survey sequences (GSS) dataset. In total, the chloroplast genomes of *L. floccosa* ssp. *floccosa* and *L. floccosa* ssp. *grandiflora* differed by seven nucleotides (five SNPs and two indels). These five SNPs were confirmed by Sanger sequencing.

Nearly 36 kbp of mitochondrial sequence data were aligned successfully between *L. floccosa* ssp. *floccosa*, *L. floccosa* ssp. *grandiflora* and the *C. papaya* complete mitochondrial genome, at an average coverage of over 100×. Within the alignment, *L. floccosa* ssp. *floccosa* and *L. floccosa* ssp. *grandiflora* differed by two nucleotides. The two SNPs were confirmed by Sanger sequencing.

Approximately 8.7 kbp of the nuclear genome was assembled and aligned from the *L. floccosa* ssp. *floccosa* and *L. floccosa* ssp. *grandiflora* datasets against the *L. alba* GSS and EST datasets. Approximately 95% of this alignment was derived from the *L. alba* GSS dataset with the remaining from the EST dataset.

Sanger sequencing of the five chloroplast loci, two mitochondrial loci and one nuclear locus containing SNPs among the five subspecies of *L. floccosa*, as well as *L. alba*, combined with previously obtained chloroplast and nuclear sequences, resulted in datasets of the following size: chloroplast, 2643 bp; mitochondrial, 303 bp; nuclear 1640 bp; total alignment, 4586 bp (Table S5; GenBank accession numbers HQ179770–HQ179849).

**Figure 2:**
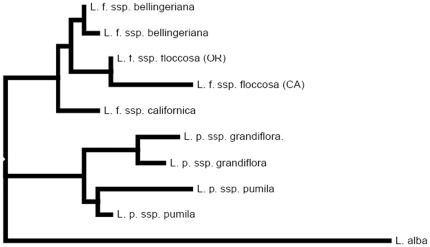
Results of the Bayesian inference of phylogeny of combined chloroplast, mitochondria and nuclear sequences. Numbers above branches indicate posterior probabilities. 4586 bp; 26 parsimony-informative sites.

The Kishino-Hasegawa and Shimodaira-Hasegawa tests found no conflict between the separate chloroplast, mitochondrial and nuclear datasets (*p* = 0.12–0.52). The combined MP strict consensus, RAxML (neither shown) and Bayesian majority rule consensus trees shared identical topology (Figure 2; Figure S3). The final phylogenetic analysis of the combined chloroplast, mitochondria and nuclear datasets resulted in a fully resolved phylogeny (all posterior probabilities of 1.0) of all *L. floccosa* subpecies.

Results of the chloroplast molecular clock analysis indicate an estimated divergence between the clades containing *L. floccosa* ssp. *floccosa* and *L. floccosa* ssp. *grandiflora* of 32.7–33.5 thousand years ago (95% Higher Posterior Densities 32.6, 32.8; 33.4, 33.6).

## Discussion

### Criterion 1

#### Species sympatry

As indicated by the results of the three year field monitoring survey, spatially and temporally *L. floccosa* ssp. *grandiflora* is sympatric with *L. floccosa* ssp. *floccosa* (Figure S1; Figure S2). This fulfills Coyne and Orr's first criterion which requires that the two taxa in question are largely or completely sympatric.

### Criterion 2

#### Reproductive isolation

With the exception of one specimen, out of the 240 wild collected *L. floccosa* ssp. *floccosa* and *L. floccosa* ssp. *grandiflora* specimens analyzed in the microsatellite analysis, all plants surveyed showed fixed differences and nearly complete homozygosity at all loci surveyed. In contrast, all artificial *L. floccosa* ssp. *floccosa*×*L. floccosa* ssp. *grandiflora* hybrids we analyzed showed a pattern of allele inheritance that would be expected if hybridization were occurring in the wild (Table S2). Overall, these results indicate that, in the wild, there is almost no evidence for genetic exchange between the two taxa. Pragmatically, this is likely due to the breeding systems of *L. floccosa* ssp. *floccosa* and *L. floccosa* ssp. *grandiflora*. The two taxa are partially (*L. floccosa* ssp. *grandiflora*) or predominately (*L. floccosa* ssp. *floccosa*) selfing [18], thus there is a natural prezygotic barrier to reproduction between the taxa.

The germination rate of the hybrid seeds was 11% (Table S1). This rate of germination is less than the 20%–25% germination rate we have typically found from field collected seeds of *L. floccosa* ssp. *floccosa* and *L. floccosa* ssp. *grandiflora* [18]. This factor, compounded with the result that over two thirds of the successful hybrid germinates were depauperate and died before maturity, indicates a high level of post zygotic reproductive isolation between the taxa.

Although one specimen of *L. floccosa* ssp. *grandiflora* collected in the wild shared an allele at one locus with *L. floccosa* ssp. *floccosa*, indicating possible natural hybridization between the species, this result conflicts with the consistent heterozygosity found in most loci of the artificial greenhouse crosses were surveyed. This result, however, could be a product of introgression (backcrossing) or retention of an ancestral allele.

Clearly, high levels of both pre and post zygotic reproductive isolation exist between *L. floccosa* ssp. *floccosa* and *L. floccosa* ssp. *grandiflora*, as measured in this analysis. We can conclude that Coyne and Orr's second criterion that substantial reproductive isolation exists, has been satisfied.

### Criterion 3

#### Sister relationship

The results of our phylogenetic analysis, derived from seven chloroplast, two mitochondria and two nuclear loci, indicate that the sister taxon to *L. floccosa* ssp. *grandiflora* is *L. floccosa* ssp. *pumila*. Additionally, the sister taxon to *L. floccosa* ssp. *floccosa* is *L. floccosa* ssp. *bellingeriana*. Therefore, *L. floccosa* ssp. *floccosa* and *L. floccosa* ssp. *grandiflora* do not strictly accord with Coyne and Orr's criterion that the taxa in question must have a sister relationship.

The clades to which *L. floccosa* ssp. *floccosa* and *L. floccosa* ssp. *grandiflora* belong could be recognized as separate, sister species. We feel, however, that this strategy would amount to a taxonomic “sleight of hand” rather than recognition of biological reality. Each subspecies within *L. floccosa* arguably represents a valid evolutionarily significant unit [12] and, in the past, each of the subspecies within this complex, with the exceptions of *L. floccosa* ssp. *californica* and *L. floccosa* ssp. *grandiflora*, have been recognized as species [19]. Furthermore, the microsatellite survey and crossing experiment conducted in this study argue that *L. floccosa* ssp. *grandiflora* could be elevated to species status. These results also possibly portend that future research may result in the elevation to species, all, or other subspecies within *L. floccosa* based on either, or both, biological and phylogenetic species concepts.

Additionally, the possibility of a sympatric speciation event involving *L. floccosa* ssp. *pumila*, versus either *L. floccosa* ssp. *floccosa* or *L. floccosa* ssp. *grandiflora*, is unlikely. This is due to the fact that all *L. floccosa* ssp. *pumila* populations are isolated on the summits of two volcanic mesa buttes (collectively known as the Table Rocks). These summits are approximately 250 meters above the valley floor where populations of *L. floccosa* ssp. *floccosa* and *L. floccosa* ssp. *grandiflora* are found (Figure S4). Likewise, all known populations of *L. floccosa* ssp. *bellingeriana* occur in a geographically and ecologically distinct region, in the foothills and mountains east of the range of *L. floccosa* ssp. *floccosa*.

While theoretically it is plausible that approximately 33,000 years ago the divergence of *L. floccosa* ssp. *floccosa* and *L. floccosa* ssp. *grandiflora* occurred by means of a sympatric speciation event, confirmation of this would require data satisfying Coyne and Orr's fourth criterion, that the existence of an allopatric phase was very unlikely. This criterion is pragmatically in this study, as in most cases, impossible to address. Coyne and Orr concede “Whether criterion 4 is satisfied usually involves a somewhat subjective judgment about the likelihood of events in the unrecoverable past.” [1] (p. 143). Moreover, because a sympatric phase between *L. floccosa* ssp. *floccosa* and *L. floccosa* ssp. *grandiflora* cannot be realistically evaluated, simply inferring allopatry risks an unknown type II error (failure to reject the null hypothesis when the alternative hypothesis is true). In addition, the choice between sympatric and allopatric speciation may not be strictly dichotomous [7]. Parapatric speciation, as well as a mixed mode of speciation, involving both allopatry and sympatry may account for speciation events such as the *L. floccosa* ssp. *floccosa* and *L. floccosa* ssp. *grandiflora* divergence.

Moreover, the divergence between *L. floccosa* ssp. *floccosa* and *L. floccosa* ssp. *grandiflora* might be explained by another controversial mode of speciation, namely reinforcement speciation. However, a study of this possibility would require currently existing allopatric and sympatric populations of both *L. floccosa* ssp. *floccosa* and *L. floccosa* ssp. *grandiflora* as well as natural populations of hybrids [20]. Currently, no allopatric populations of *L. floccosa* ssp. *grandiflora* or natural populations of *L. floccosa* ssp. *floccosa*×*L. floccosa* ssp. *grandiflora* hybrids are known to exist [18].

Although we debate the efficacy of Coyne and Orr's fourth criterion, criteria one through three do provide a much needed and logical framework around which to study putative sympatric speciation events. Given the current and likely forthcoming controversy of sympatric speciation, future studies should and will likely be required to undertake rigorous analyses and experimental evaluations. As demonstrated in this study, recent technological advances, such as next generation sequencing, may allow the recovery of fully resolved phylogenies among recently diverged plant taxa. Except in examples in which the evolutionary history of the taxa in question are complex, for example due to much reticulation, these data will likely be necessary to provide the rigorous and uncontroversial proofs necessary to either prove or disprove putative sympatric speciation events.

## Methods

### Ethics statement

All necessary permits were obtained for the described field studies. Permission was granted from the Nature Conservancy to conduct this study and collect plant material located on property owned by the organization. Although *L. floccosa* ssp. *grandiflora* is listed as an endangered species by the United State Fish and Wildlife Service, and the State of Oregon, because this field study was conducted on private land no government issued permits were required. Plant material from the remaining taxa were obtained from plants grown from germplasm accessions obtained from the Arid Land Plant Genetic Resources Unit (Parlier, California, USA).

### Spatial and temporal sympatry

Over the course of three flowering seasons (between early March and late May, 2006–2008), eight vernal pools in which both *L. floccosa* ssp. *floccosa* and *L. floccosa* ssp. *grandiflora* populations reside were monitored. In total, 183 one meter square plots and approximately 1000 plants (∼400 *L. floccosa* ssp. *floccosa* and ∼600 *L. floccosa* ssp. *grandiflora* individuals) were surveyed. At the beginning of each field season, the distance of each *Limnanthes* individual from the edge of the vernal pool was measured. On a bi-weekly basis, within each plot, the flowering stages of all *Limnanthes* individuals were recorded (in bud, flowering, in seed).

### Reproductive isolation

From the 389 *Limnanthes* microsatellite primers designed by Kirshore et al. [21], we picked twelve based on allele length and whether those alleles differentiated the samples of *L. floccosa* ssp. *floccosa* and *L. floccosa* ssp. *grandiflora* tested in that study. In total, 120 individuals from each taxon, collected from three vernal pools (40 each vernal pool), during one season, were surveyed. Microsatellite forward primers were modified by a 5′ concatenation of the 18-mer 5′-TGTAAAACGACGGCCAGT-3′. This permitted concurrent fluorescence labeling of PCR products by a third primer with an incorporated fluorophore. Amplicons were obtained following the polymerase chain reaction (PCR) protocol of Schuelke [22]. PCR products were resolved in an ABI 3730 capillary DNA sequencer. Electropherograms were analyzed using ABI GeneMapper software.

In a greenhouse experiment, 40 individuals of *L. floccosa* ssp. *floccosa* were crossed by hand with 40 individuals of *L. floccosa* ssp. *grandiflora*, in both directions (80 crosses total). These plants were grown from seed collected during one season, in the wild, from three vernal pools located within a five kilometer radius. Seeds produced from those crosses were germinated following the protocol of Toy and Willingham [23]. All putative hybrid plants surviving to maturity were genotyped using the microsatellite primers described above.

### Phylogeny

DNA was obtained from fresh leaves, collected in the wild or plants grown from germplasm accessions obtained from the Arid Land Plant Genetic Resources Unit (Parlier, California, USA). Seeds from the accessions were germinated following Toy and Willingham [23]. Seedlings were grown in a greenhouse until maturity and keyed using Ornduff [24] to confirm their taxonomic identity. Voucher specimens have been placed in the Oregon State University Herbarium (OSC). Approximately 50 mg of plant material were used to extract DNA using a DNeasy Plant Mini kit.

Illumina DNA preparation and amplification followed the protocol of Meyers and Liston [25], replacing the 3 bp 5′ tags with the 4 bp tags CACT and GGGT. Individual libraries of *L. floccosa* ssp. *floccosa* and *L. floccosa* ssp. *grandiflora* were pooled into a 2× multiplex sequencing library. A total of 10 pmol was used for 76 cycles of paired-end sequencing in a single lane of an Illumina GAI in the Central Service Laboratory of the Oregon State University Center for Genome Research and Biocomputing. Assembly of the microreads followed a modified protocol of Whittall et al. [26]. Microreads, after tag removal, were assembled into contigs with the *de novo* assembler Velvet v. 0.7 [27]. These *de novo* contigs were aligned to a complete *Carica papaya* chloroplast genome (GenBank NC010323) using the alignment program Mulan [28]. The consensus sequence of aligned contigs was next merged with the *C. papaya* reference sequence to form a chimeric pseudo-reference. The pseudo-reference was composed of approximately 95% *de novo* sequence and 5% *C. papaya* reference sequence where gaps in the *de novo* sequence were initially found. The original microreads from each taxon were next re-aligned against the pseudo-reference using the reference guided assembler RGA (http://rga.cgrb.oregonstate.edu/) using a minimum depth of 2×, a maximum allowable error/mismatch of 0.03 and a 70 percent majority minimum for SNP acceptance. PCR primers were designed using Primer3 [29] for remaining gaps, areas with long lengths of repetitive DNA and putative SNPs. The subsequent amplification products were Sanger sequenced.

*De novo* contigs were also aligned to a *C. papaya* complete mitochondrial genome (GenBank NC012116), a *L. alba* GSS dataset (GenBank DX504195–DX507856) and a *L. alba* EST dataset (GenBank FD644000–FD656247) using RGA. Additionally, large portions of the *L. alba* chloroplast were assembled with Mulan, from the GSS dataset sequences, using the *L. floccosa* ssp. *grandiflora* chloroplast genome as a reference template.

From the resulting alignments, eight sets of PCR primers (five chloroplast, two mitochondrial and one nuclear) were designed using Primer3 [29] to amplify regions containing putative SNPs among *L. floccosa* ssp. *floccosa*, *L. floccosa* ssp. *grandiflora* and *L. alba*. In turn, we sequenced those eight regions from one additional sample of *L. floccosa* ssp. *floccosa* and *L. floccosa* ssp. *grandiflora* plus two samples each of all remaining subspecies within *L. floccosa*. Although selecting these SNPs to analyze in the remaining *L. floccosa* taxa raises the question of ascertainment bias, the paucity of genetic variation found in a previous study [16], as well as this study, particularly within the chloroplast genomes, leads us to believe that the potential for this error is minimal. Furthermore, this phylogeny is based most heavily on data obtained from chloroplasts, which within these recently diverged taxa, are maternally inherited and do not recombine, thereby also lessening the potential for ascertainment bias.

Following PCR, all products were purified using QIAquick PCR purification kits. All Sanger sequencing was performed by the High-Throughput Genomic Unit (Seattle, Washington, USA). Sequences were aligned “by eye” and analyzed using BioEdit for Windows 95/98 [30]. The resulting datasets were combined with additional chloroplast and nuclear ribosomal DNA ITS sequences obtained from the same samples in a previous study [13] (GenBank accessions FJ895938–FJ895944, FJ895947–FJ895949, FJ895993–FJ895995, FJ895982–FJ895991, FJ895912–FJ895914, FJ895915–FJ895919, FJ895906–FJ895907).

To test whether individual chloroplast, mitochondria and nuclear datasets should be combined, Kishino-Hasegawa and Shimodaira-Hasegawa tests [31], [32] using PAUP* were employed.

Phylogenetic analyses were conducted using PAUP* version 4b10, RAxML [33] and MrBayes version 3.1.2 [34]. For individual and combined datasets, most parsimonious trees were found using branch and bound maximum parsimony (MP) searches within PAUP*, employing the furthest addition sequence setting and MulTrees on. Gaps were scored as missing data. Branch support was assessed using 1000 bootstrap replicates.

Modeltest 3.7 [35] was used to select the model rate that best fit each dataset. An F81 model was selected for the chloroplast and mitochondria datasets and a Tamura Nei model incorporating a gamma distribution (TrN+gamma) for the nuclear data set. These models were set for all Bayesian searches, except because a TrN+gamma model is not available in MrBayes, model parameters for the nuclear dataset were estimated by simplifying the general time reversible (GTR) model. Likewise, a GTR+gamma model was set for the RAxML (ML) search. Bayesian searches were conducted in one run using one cold and three heated Markov Chains, over two million generations, sampling every 100 generations. All trees generated within the burn-in period (2,000 generations) were discarded and posterior probability confidence values were based only on trees found in the stationary phase.

A divergence date between the clades containing *L. floccosa* ssp. *floccosa* and *L. floccosa* ssp. *grandiflora* was calculated using BEAST v1.5.4. Analyses were run for ten million generations, with parameters sampled every 1000 generations, using a GTR model for both the combined chloroplast and nuclear datasets. Substitution rates used were based on previous molecular clock calculations for plant chloroplasts (2.2–2.8×10^−9^ substitutions per site per year [36]). Results of this analysis were visualized using Tracer v1.4.

## Supporting Information

Table S1 **Results of the greenhouse hybridization experiment between** ***L. floccosa*** **ssp.** ***floccosa*** **and** ***L. floccosa*** **ssp.** ***grandiflora*** **.**
(DOC)

Table S2 **Results of microsatellite analysis of 240** ***L. f.*** **ssp.** ***floccosa*** **and** ***L. f.*** **ssp.** ***grandiflora*** **plants collected in the wild and hybrids between the species.** Loci numbers refer to the numbering scheme of Kirshore et al. (2004). All alleles at 1.0 frequency unless noted otherwise in parentheses.
(DOC)

Table S3 **Total number of genomic paired-ends reads and base pairs, for each taxon, generated from the Illumina run.** The percent coverage is based on an estimated genome size of 1.36 gigabases.
(DOC)

Table S4 **Total number of chloroplast paired-end reads, base pairs, percentage of chloroplast reads within total genomic dataset and coverage depth.**
(DOC)

Table S5 **Loci sequenced for the phylogenetic analysis of** ***L. floccosa*** **.** *Indicates sequences obtained in a previous study^15^. ∧ Indicates that the locus sequenced contained one SNP and one indel found within the chloroplast.
(DOC)

Figure S1 **Plot of distances of** ***L. f.*** **ssp.** ***floccosa*** **and** ***L. f.*** **ssp.** ***grandiflora*** **plants from the edge of the vernal pools near which they co-occur.** (*L. f.* ssp. *floccosa* S.D. 1.2; *L. f.* ssp. *grandiflora* S.D. 0.7)
(TIF)

Figure S2 **Bud, flower and seed timing of sympatric populations of** ***L. f.*** **ssp.** ***floccosa*** **and** ***L. f.*** **ssp.** ***grandiflora*** **.** Day numbers indicate time period between early March and late May.
(TIF)

Figure S3 **Results of the Bayesian inference of phylogeny of chloroplast, mitochondria and datasets.** Numbers above branches indicate posterior probabilities.
(TIF)

Figure S4 **Upper Table Rock, Oregon, USA.** One of two volcanic mesa buttes on which populations *L. f.* ssp. *pumila* are located.
(TIF)
