# Supplementary material for: An Evaluation of Putative Sympatric Speciation within Limnanthes (Limnanthaceae)
Source: PLoS One. 2012 May 1;7(5):e36480. doi: 10.1371/journal.pone.0036480 (PMC3341363; doi:10.1371/journal.pone.0036480)
Supplement: Table S1 — Results of the greenhouse hybridization experiment between L. floccosa ssp. floccosa and L. floccosa ssp. grandiflora . (DOC) [file pone.0036480.s001.doc]

**Table S1. Results of the greenhouse hybridization experiment between *L. floccosa* ssp. *floccosa* and *L. floccosa* ssp. *grandiflora*.**

| **Cross** | **Number of seeds produced** | **Number of seeds germinated** | **Number of plants surviving to flowering** | **Number of fertile individuals** |
| --- | --- | --- | --- | --- |
| *L. f.* ssp*. floccosa* (maternal) × *L. f.* ssp. *grandiflora* (paternal) n=40 | 115 | 16 | 5 | 0 |
| *L. f.* ssp. *grandiflora*  (maternal) × *L. f.* ssp. *floccosa* (paternal) n=40 | 154 | 14 | 4 | 0 |
