# Supplementary material for: An Evaluation of Putative Sympatric Speciation within Limnanthes (Limnanthaceae)
Source: PLoS One. 2012 May 1;7(5):e36480. doi: 10.1371/journal.pone.0036480 (PMC3341363; doi:10.1371/journal.pone.0036480)
Supplement: Table S2 — Results of microsatellite analysis of 240 L. f. ssp. floccosa and L. f. ssp. grandiflora plants collected in the wild and hybrids between the species. Loci numbers refer to the numbering scheme of Kirshore et al. (2004). All alleles at 1.0 frequency unless noted otherwise in parentheses. (DOC) [file pone.0036480.s002.doc]

Table S2. **Results of microsatellite analysis of 240 *L. f.* ssp. *floccosa* and *L. f.* ssp. *grandiflora* plants collected in the wild and hybrids between the species.** Loci numbers refer to the numbering scheme of Kirshore et al. (2004). All alleles at 1.0 frequency unless noted otherwise in parentheses.

| **Locus** | **Allele lengths (bp)**  ***L. f.* ssp. *floccosa*; n=120** | **Allele lengths (bp)**  ***L. f.* ssp. *grandiflora*; n=120** | **Heterozygous in *floccosa* × *grandiflora* hybrids n=8** | **Comments** |
| --- | --- | --- | --- | --- |
| 53 | 336 | 342 | no | maternally inherited |
| 82 | 243 | 247 | yes |  |
| 96 | 311 | 325 | no | maternally inherited |
| 99 | 191 | 197 | yes |  |
| 167 | 245 | 255 | yes |  |
| 168 | 172 | 160 | yes |  |
| 187 | 263 | 266 | yes |  |
| 318 | 383 | 373 (0.99) , 383 (0.01) | yes | One heterozygous *grandiflora* individual with allele 383 |
| 322 | 461 (0.64), 463 (0.36) | 419 | yes |  |
| 427 | 277 | 283 | no | maternally inherited |
| 572 | 352 | 362 | yes |  |
| 583 | 188 | 182 | no | maternally inherited |
