# Supplementary material for: An Evaluation of Putative Sympatric Speciation within Limnanthes (Limnanthaceae)
Source: PLoS One. 2012 May 1;7(5):e36480. doi: 10.1371/journal.pone.0036480 (PMC3341363; doi:10.1371/journal.pone.0036480)
Supplement: Table S3 — Total number of genomic paired-ends reads and base pairs, for each taxon, generated from the Illumina run. The percent coverage is based on an estimated genome size of 1.36 gigabases. (DOC) [file pone.0036480.s003.doc]

**Table S3.** **Total number of genomic paired-ends reads and base pairs, for each taxon, generated from the Illumina run.** The percent coverage is based on an estimated genome size of 1.36 gigabases

|  | **Paired-end reads** | **Total base pairs** | **Coverage** |
| --- | --- | --- | --- |
| *L. floccosa* ssp. *floccosa* | 5,071,991 | 703,366,704 | 0.537 |
| *L. floccosa* ssp. *grandiflora* | 4,925,217 | 709,231,248 | 0.521 |
