# Supplementary material for: An Evaluation of Putative Sympatric Speciation within Limnanthes (Limnanthaceae)
Source: PLoS One. 2012 May 1;7(5):e36480. doi: 10.1371/journal.pone.0036480 (PMC3341363; doi:10.1371/journal.pone.0036480)
Supplement: Table S4 — Total number of chloroplast paired-end reads, base pairs, percentage of chloroplast reads within total genomic dataset and coverage depth. (DOC) [file pone.0036480.s004.doc]

**Table S4. Total number of chloroplast paired-end reads, base pairs, percentage of chloroplast reads within total genomic dataset and coverage depth.**

|  | **Paired-end reads** | **Total base pairs** | **% of total reads** | **Coverage depth** |
| --- | --- | --- | --- | --- |
| *L. floccosa* ssp. *floccosa* | 698,606 | 50,299,632 | 6.88 | 335X |
| *L. floccosa* ssp. *grandiflora* | 713,779 | 51,392,088 | 7.25 | 343X |
