# Supplementary material for: An Evaluation of Putative Sympatric Speciation within Limnanthes (Limnanthaceae)
Source: PLoS One. 2012 May 1;7(5):e36480. doi: 10.1371/journal.pone.0036480 (PMC3341363; doi:10.1371/journal.pone.0036480)
Supplement: Table S5 — Loci sequenced for the phylogenetic analysis of L. floccosa . *Indicates sequences obtained in a previous study15. ∧ Indicates that the locus sequenced contained one SNP and one indel found within the chloroplast. (DOC) [file pone.0036480.s005.doc]

**Table S5. Loci sequenced for the phylogenetic analysis of *L. floccosa*.** *Indicates sequences obtained in a previous study^15^. ^ Indicates that the locus sequenced contained one SNP and one indel found within the chloroplast.

| **Locus** | **Number of base pairs** | **Primer sequences** |
| --- | --- | --- |
| **Chloroplast** |  |  |
| *psb*A - *trn*H | 217 | 5’-aatccactgccttgatccac-3’  5’-cgtgctaaccttggtatggaa-3’ |
| *psb*Z - *trn*G | 384 | 5’-aatgacccctcccacaattt-3’  ‘5’-ggttcatgcatgtttgttgc-3’ |
| *trn*K - *rps*16 | 86 | 5’-ttccacaccgagaattcaaa-3’  5’-tccattttgattattatatccatgttt-3’ |
| *rpl*32 – *trn*L^ | 420 | 5’-tccaaaaagcgtgttcgtaa-3’  5’-cgtaattggtcggggttttt-3’ |
| *ycf*1^ | 393 | 5’-gcccgagatatcaaatgaa-3’  5’-aatgttgttatcggtataactgagtc-3’ |
| *trn*L* | 401 | 5’-cgaaatcggtagacgctacg-3’  5’-ggggatagagggacttgaac-3’ |
| *trn*S – *trn*G* | 742 | 5’-agatagggattcgaaccctcggt-3’  5’-gtagcgggaatcgaacccgcatc-3’ |
| **Mitochondria** |  |  |
| *nad*7 | 81 | 5’-tacgtcaggatccgattggt-3’  5’-cgggcctaagtgaaagtgaa-3’ |
| *nadh*1 | 222 | 5’-gaggtgcagggaaggagag-3’  5’-acaactaatatctttactggctagagg-3’ |
| **Nuclear** |  |  |
| unknown function | 969 | 5’-gcatttgctccggatc-3’  5’-tggtatgatcgcatc-3’ |
| nrITS* | 671 | 5’-ggaaggagaagtcgtaacaagg-3’  5’-tatgcttaaactcagcgggt-3’ |
