# Supplementary figures and images for: An Evaluation of Putative Sympatric Speciation within Limnanthes (Limnanthaceae)
Source: PLoS One. 2012 May 1;7(5):e36480. doi: 10.1371/journal.pone.0036480 (PMC3341363; doi:10.1371/journal.pone.0036480)

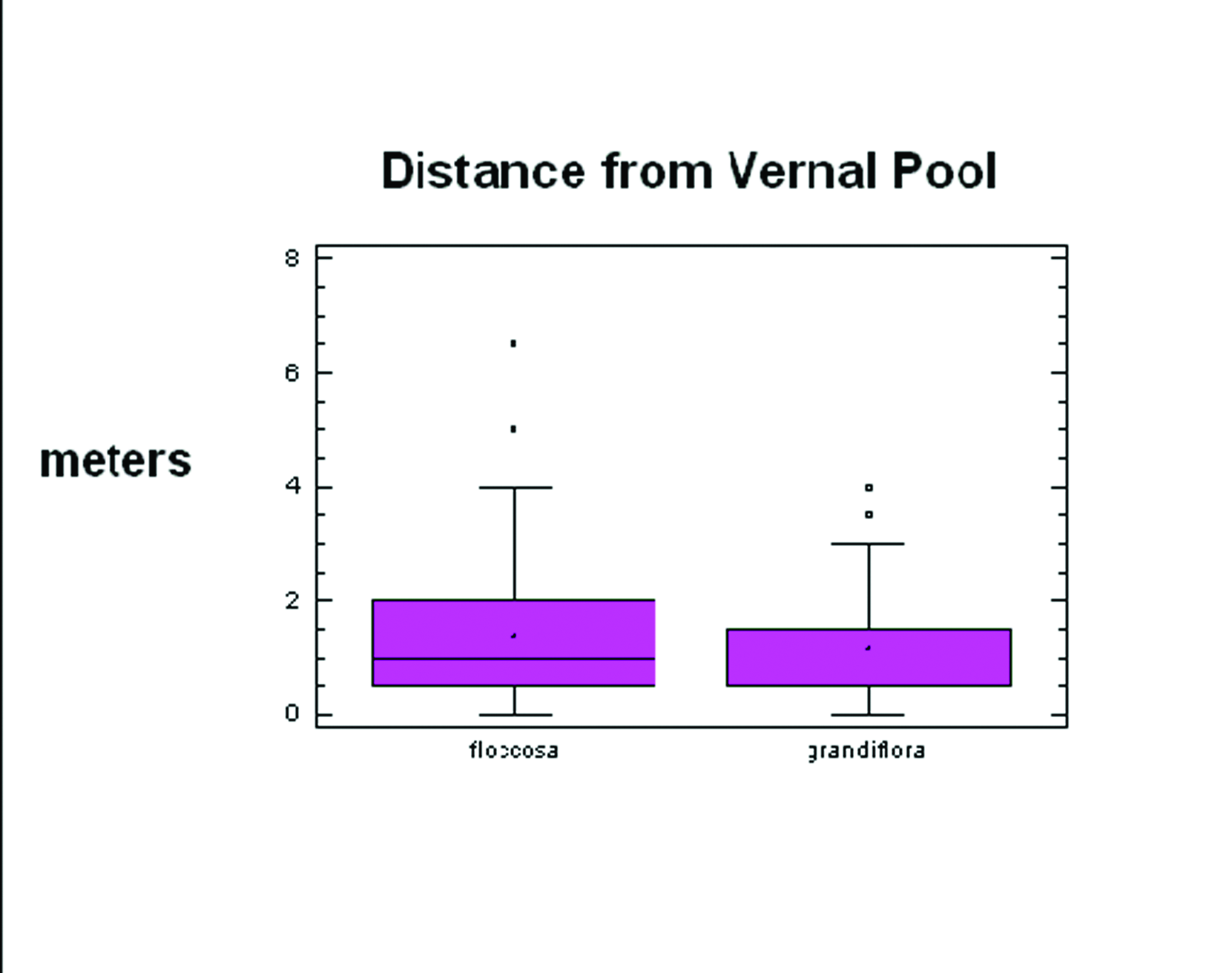

Supplement: Figure S1 — Plot of distances of L. f. ssp. floccosa and L. f. ssp. grandiflora plants from the edge of the vernal pools near which they co-occur. (L. f. ssp. floccosa S.D. 1.2; L. f. ssp. grandiflora S.D. 0.7) (TIF) [file pone.0036480.s006.tif]

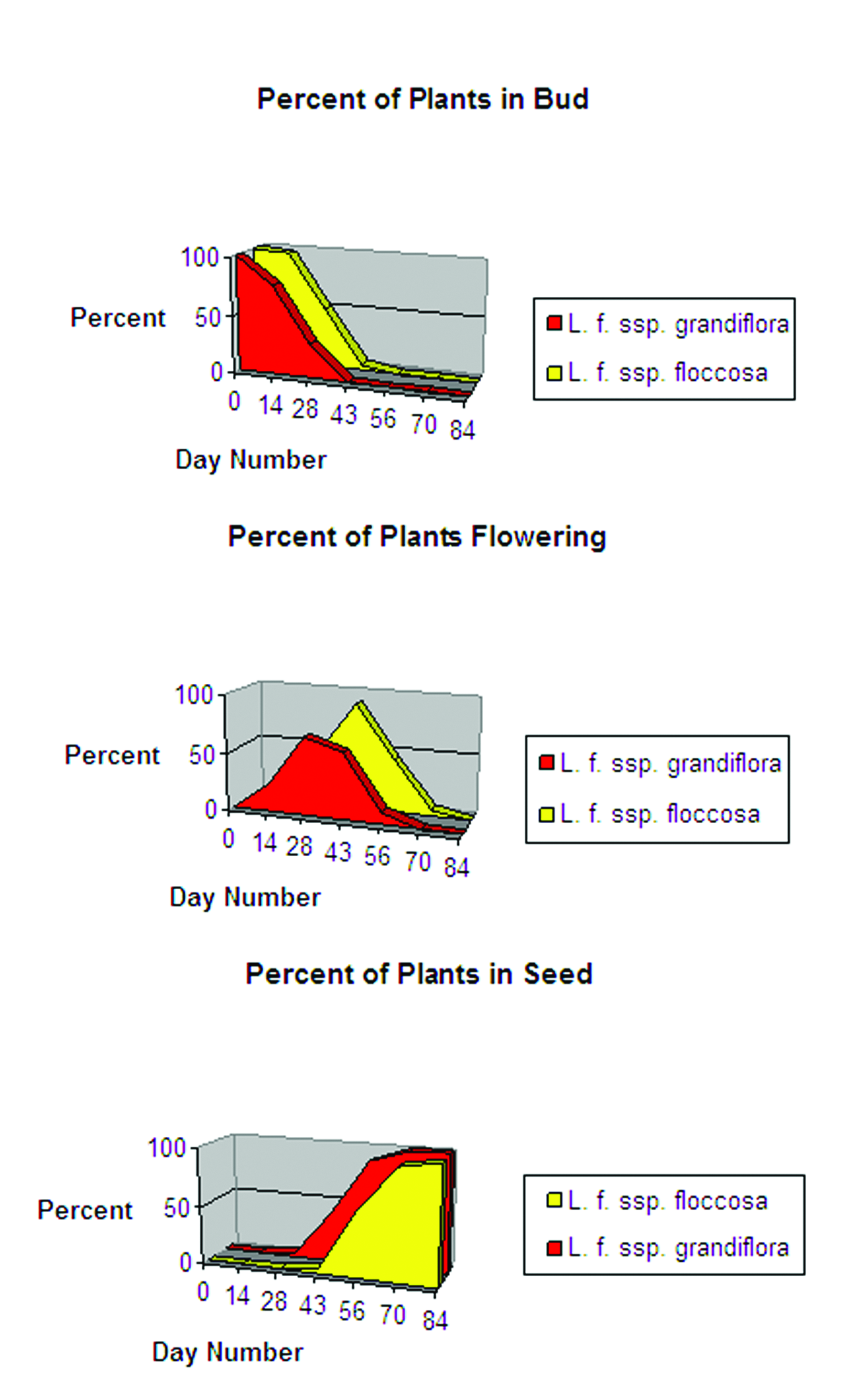

Supplement: Figure S2 — Bud, flower and seed timing of sympatric populations of L. f. ssp. floccosa and L. f. ssp. grandiflora . Day numbers indicate time period between early March and late May. (TIF) [file pone.0036480.s007.tif]

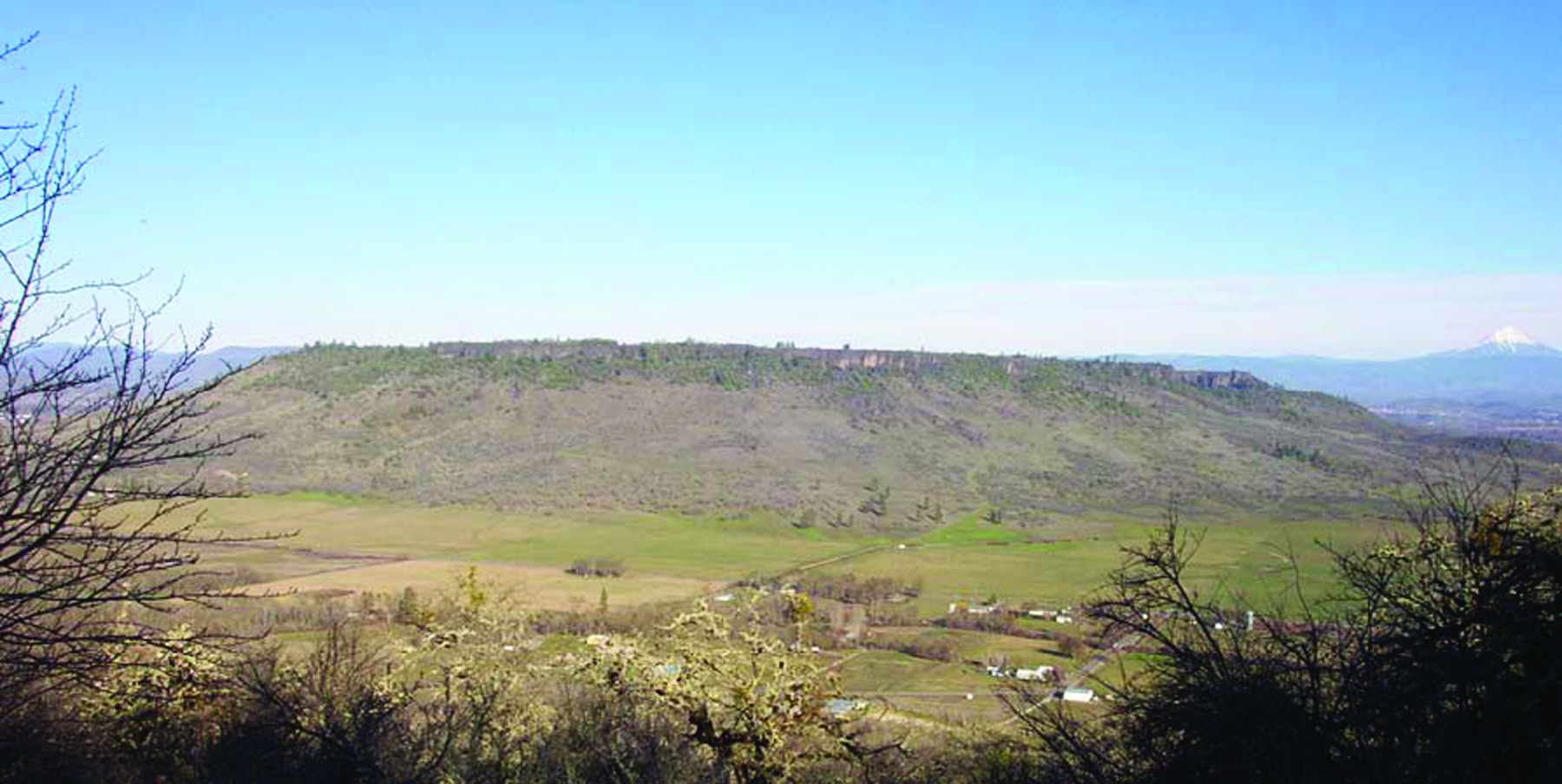

Supplement: Figure S4 — Upper Table Rock, Oregon, USA. One of two volcanic mesa buttes on which populations L. f. ssp. pumila are located. (TIF) [file pone.0036480.s009.tif]
